# Neuroprotective effects of human umbilical cord mesenchymal stem cells (Neuroncell-EX) in a rat model of ischemic stroke are mediated by immunomodulation, blood–brain barrier integrity, angiogenesis, and neurogenesis

**DOI:** 10.1007/s11626-025-01037-y

**Published:** 2025-05-13

**Authors:** Sze-Piaw Chin, Erlena Nor Asmira Abd.Rahim, Natasha Najwa Nor Arfuzir

**Affiliations:** 1 Cytopeutics Sdn Bhd, Bio-X Centre, Persiaran Cyberpoint Selatan, Cyber 8, 63000 Cyberjaya, Selangor Malaysia; 2 CMH Specialist Hospital, Jalan Tun Dr. Ismail, 70200 Seremban, Negeri Sembilan Malaysia; 3 M.Kandiah Faculty of Medicine and Health Sciences, Universiti Tunku Abdul Rahman (UTAR), Bandar Sungai Long, 43000 Kajang, Selangor Malaysia

**Keywords:** hUC-MSCs, Ischemic stroke, Neuroinflammation, Immunomodulation, Neurogenesis, Angiogenesis

## Abstract

Human umbilical cord–derived mesenchymal stem cells (hUC-MSCs) are a potential off-the-shelf product for acute ischemic stroke. This study explored the underlying mechanism of Cytopeutics® hUC-MSCs (Neuroncell-EX) as well as its feasibility and efficacy at two different doses: 2 × 10^6^ cells per rat and 4 × 10^6^ cells/rat in middle cerebral artery occlusion (MCAO) ischemic stroke model for 28 d. Modified neurological severity score (mNSS) and rotarod tests were evaluated at days 1, 4, 7, and 14. Transforming growth factor-beta 1 (TGF-β1), interleukin-1 receptor antagonist (IL-1Ra), and vascular endothelial growth factor (VEGF) were evaluated by enzyme-linked immunosorbent assay (ELISA) at days 4 and 28. Immunohistochemistry expression of aquaporin-4 (AQP4) and neuronal protein marker (NeuN) were performed at days 4 and 28, respectively. Both doses of Neuroncell-EX showed significant lower mNSS scores at days 7 and 14 compared to stroke control. Both Neuroncell-EX groups showed significant longer latency time at day 7, with only 4 × 10⁶ cells/rat group having significant longer time at day 14 than stroke control. At both time points, the 2 × 10⁶ cells/rat group had significantly higher TGF-β1 and IL-1Ra levels, with significantly increased TGF-β1 only observed in 4 × 10⁶ cells/rat group at day 4 compared to stroke control. The VEGF levels were significantly lower at day 4 but then significantly increased at day 28 in both Neuroncell-EX groups than stroke control. AQP4 expression was significantly higher in stroke control compared to healthy control at day 4. Both doses of Neuroncell-EX showed significantly higher NeuN expression compared to stroke control at day 28. There is a weak correlation between TGF-β1 with VEGF and inversely with AQP4. These results suggest that Neuroncell-EX is feasible and effective in promoting functional recovery and neuroprotection in ischemic rats, potentially through immunomodulation, angiogenesis, and neurogenesis mechanisms.

## Introduction

Stroke is a medical condition caused by an interruption in blood supply to the brain, resulting in cell death. To date, approximately 13.7 million people worldwide are diagnosed with stroke each year (Kuriakose & Xiao 2020). Approximately 87% of stroke cases are ischemic strokes, leading to symptoms such as sudden numbness or weakness in the face, arm, or leg, as well as difficulty speaking and walking (Li *et al*. 2021a, b). The loss of blood flow to the brain sets off an ischemic cascade within seconds to minutes, triggering a series of biochemical events. These events include the activation and infiltration of inflammatory cells, and the production of pro-inflammatory mediators that eventually lead to blood–brain barrier (BBB) disruption, neuronal injury, and vascular remodeling (Lakhan *et al*. 2009). Moreover, prolonged cerebral ischemia could cause long-term neuronal damage through excitotoxicity and ionic imbalance, microglia-mediated neuroinflammation, oxidative stress, loss of cellular energy demand, and BBB dysfunction (Ahad *et al*. 2020).

Thrombolytic therapy with recombinant tissue plasminogen activator (rt-PA) is an endorsed acute ischemic stroke therapy (Mijajlovic 2014) which dissolves blood clots and restores vascularization. However, the limitation of this therapy is the narrow therapeutic time window of 3–4.5 h after stroke onset which leaves a large number of stroke patients ineligible for this “gold standard” treatment (Mijajlovic 2014).

In animal studies of stroke, the middle cerebral artery occlusion (MCAO) model is preferred because it closely mimics human ischemic stroke, particularly middle cerebral artery infarction, which is the most common type of stroke in humans (Fluri *et al*. 2015). In clinical relevance, the middle cerebral artery (MCA) supplies a large portion of the cerebral cortex, including the motor and sensory areas (Valante *et al*. 2015). This model is widely used to investigate potential treatment effects, as it is a well-established and standardized animal model.

The application of mesenchymal stem cells (MSCs) as a potential immunomodulatory and neuroprotective therapy has garnered considerable attention in stroke management due to their demonstrated safety and efficacy in previous preclinical and clinical studies (Chrostek *et al*. 2019; Li *et al*. 2020; Ouyang *et al*. 2020). The neuroprotective effects of hUC-MSCs in stroke are most likely mediated through the reduction of inflammation following ischemic injury (Lakhan *et al*. 2009; Lin *et al*. 2011; Oh *et al*. 2018). Recent approaches have been investigated to determine the efficacy of different types of MSCs, including bone marrow (BM-MSC), adipose tissue (AT-MSC), and human umbilical cord (hUC-MSCs), for the treatment of ischemic stroke (Li *et al*. 2021a, b; Merino-González *et al*. 2016). Allogeneic hUC-MSCs offer greater advantages because they are readily available with low immunogenicity (Li *et al*. 2021a, b) whereas, autologous MSCs are hardly used in stroke management due to their longer production time and variable quality (Li *et al*. 2021a, b). Different umbilical cord sources, as well as differences in culture and expansion methods, can lead to MSC heterogeneity. Li and his co-workers observed that functional heterogeneity exists within the same tissue sample, even when subjected to identical culture methods. As a consequence, this might affect the results of stem cell therapy (Li *et al*. 2023a, b). Thus, it is very important to establish the feasibility and efficacy of our own hUC-MSCs (Neuroncell-EX) with their underlying mechanism in the disease model prior to embarking on clinical trials.

The route of administration, treatment time window, and dose of MSC administration are also critical factors in treating ischemic stroke (Jolien De *et al*. 2018). We have previously determined that hUC-MSCs exerted their anti-inflammatory effects in a dose-dependent manner via paracrine action (Chin *et al*. 2020; Tai *et al*. 2023). In this study, we investigated two doses of hUC-MSCs and the relationship of possible mechanisms of neuroprotection including systemic inflammation, angiogenesis, neurogenesis, and maintaining BBB integrity.

## Materials and method

### Cytopeutics® human umbilical cord mesenchymal stem cells (Neuroncell-EX)

The Cytopeutics® hUC-MSCs (Neuroncell-EX) product is manufactured in a clean room laboratory (Cyberjaya, Malaysia) that has been certified as compliant with Good Manufacturing Practice (GMP). After the birth of a full-term, healthy baby, umbilical cord samples were obtained with written consent from both parents. A thorough screening for genetic mutations, infections, cancers, and inherited diseases across three generations—newborns, parents, and grandparents—is conducted prior to laboratory processing. Cell processing and isolation procedures were performed based on our previous work (Chin *et al*. 2020).

### Animals

**Figure 1.**
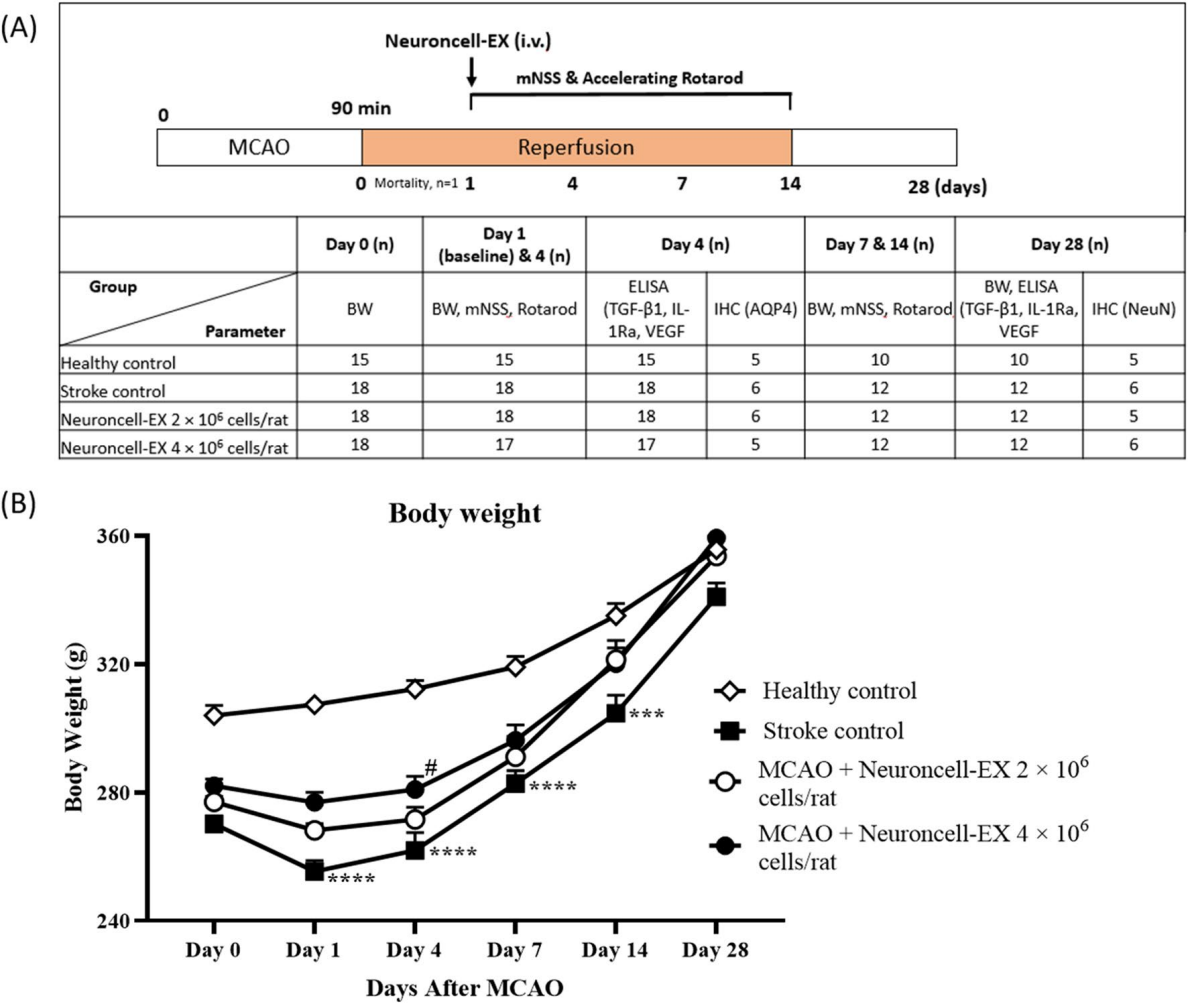
(*A*) Schematic experimental procedure with the number of animals per group for each assessment. (*B*) Time course of body weight changes exposed to 90 min of MCAO until the end of the study. Two-way ANOVA followed by post hoc Dunnett’s test was performed to compare multiple groups. Data are presented as mean ± SEM where *** indicates *p* < 0.001, **** indicates *p* < 0.0001 (stroke control versus healthy control), and *#* indicates *p* < 0.05 (Neuroncell-EX-treated groups versus stroke control). *AQP4*, aquaporin-4; *BW*, body weight; *ELISA*, enzyme-linked immunosorbent assay; *IHC*, immunohistochemistry; *IL-1Ra*, interleukin-1 receptor antagonist; *IV*, intravenous; *mNSS*, modified neurological severity score; *MCAO*, middle cerebral artery occlusion; *NeuN*, neuronal protein marker; *TGF-β1*, transforming growth factor-beta 1; *VEGF*, vascular endothelial growth factor; *SEM*, standard error of the mean.

All the animal procedures were conducted according to the Institutional Animal Ethics Committee approval with the reference number Syngene/IAEC/1324–12–2021 and reported according to the Animal Research Reporting of In Vivo Experiments (ARRIVE) guidelines. Sixty-nine (*n* = 69) male Sprague–Dawley rats (age 10–11 wk old, weight 260–320 g) were obtained from Envigo (Netherlands). The rats were quarantined for 1 wk and then acclimatized for 3–5 d before the initiation of the experiment. The rats were housed in a controlled environment with 22 ± 3 °C temperature, 50 ± 20% humidity, a light/dark cycle of 12 h each, and 15–20 fresh air changes per h, and were fed ad libitum, with irradiated laboratory rodent diet (Altromin Spezialfutter, Germany, Diet 1324) and reverse osmosis filtered drinking water. At first, rats were randomized based on their body weight into four groups consisting of (i) healthy control group (*n* = 15) receiving saline injection; (ii) stroke control group (*n* = 18) with MCAO rats receiving saline injection; (iii) MCAO rats receiving Neuroncell-EX 2 × 10^6^ cells/rat (*n* = 18); and (iv) MCAO rats receiving Neuroncell-EX 4 × 10^6^ cells/rat (*n* = 18). However, the number of rats then varied in all assessments, as written in the following study design in Fig. 1*A*. For body weight, modified neurological severity scores (mNSS), and accelerated rotarod assessments, the number of rats assessed in each group was as follows: healthy control (*n* = 15), stroke control (*n* = 18), MCAO rats treated with Neuroncell-EX 2 × 10^6^ cells/rat (*n* = 18), and MCAO rats treated with Neuroncell-EX 4 × 10^6^ cells/rat (*n* = 17) on days 1 and 4. On days 7 and 14, the groups assessed included healthy control (*n* = 10), stroke control (*n* = 12), MCAO rats treated with Neuroncell-EX 2 × 10^6^ cells/rat (*n* = 12), and MCAO rats treated with Neuroncell-EX 4 × 10^6^ cells/rat (*n* = 12). While for immunohistochemical (IHC) assessments, the number of rats assessed in AQP4 staining at day 4 was as follows: healthy control (*n* = 5), stroke control (*n* = 6), MCAO rats treated with Neuroncell-EX 2 × 10^6^ cells/rat (*n* = 6), and MCAO rats treated with Neuroncell-EX 4 × 10^6^ cells/rat (*n* = 5). On day 28 of assessing NeuN, the groups assessed included healthy control (*n* = 5), stroke control (*n* = 6), MCAO rats treated with Neuroncell-EX 2 × 10^6^ cells/rat (*n* = 5), and MCAO rats treated with Neuroncell-EX 4 × 10^6^ cells/rat (*n* = 6).

### Rat model of middle cerebral artery occlusion

Rats were subjected to MCAO using an intraluminal technique as described previously (Doǧan *et al*. 1998). On day 0, rats were initially anesthetized with 4% isoflurane (1.5–2% maintenance), and the temperature was maintained using a heating pad at 37 ± 1 °C. Rats were placed on the dorsal surface; the neck area was depilated, and the surgical area was disinfected. A median incision was made in the neck skin; the right common carotid artery was exposed. A 4–0 monofilament nylon suture was inserted from the external carotid artery into the internal carotid artery until the tip occludes the origin of the middle cerebral artery. After 90 min of middle cerebral artery occlusion, reperfusion was achieved by the withdrawal of monofilament. The rats were allowed to recover from the anesthesia on a warming pad and returned to their home cage. The healthy control group was subjected to a similar procedure without the monofilament suture insertion.

### Dosage calculation and selection of Neuroncell-EX

Neuroncell-EX cell suspension at the dose of 2 × 10^6^ cells/rat and 4 × 10^6^ cells/rat in 800 µL saline was administered by intravenous (IV) slow bolus injection into a lateral tail vein using a 24-gauge needle (24 G), at day 1 after the MCAO surgery, as shown in Fig. 1*A*. The healthy control and stroke control rats received equivalent saline without cell injection. The Neuroncell-EX dosages were estimated based on allometric scaling using a body surface area normalization from animals to humans, which converts to the human equivalent dose (HED) of 1.12 × 10^6^ cells/kg BW and 2.23 × 10^6^ cells/kg BW in humans (Nair & Jacob 2016).

### Assessment of adverse effects, morbidity, and mortality

The body weight and daily behavior for all groups were observed individually and recorded every day for 28 d post-MCAO induction.

### Neurological function assessments

Neurological function assessments were performed using modified neurological severity scores (mNSS) and accelerated rotarod task at days 1, 4, 7, and 14 after MCAO induction by an investigator blinded to the experimental groups. Rats were trained prior to MCAO induction and all tests were performed between 9.00 am to 4.00 pm. Brief procedures are described below.

### Evaluation using mNSS

A set of mNSS was applied to evaluate the severity of neurological deficits and recovery, which reflected the motor and sensory systems, reflexes, and balance functions. The assessment was conducted on days 1, 4, 7, and 14 post-MCAO induction. The scoring system was graded on a scale of 0 to 18 and was divided into three main sections including postural reflex, placing test (visual and tactile placing), and proprioception. One score point was given for the inability to perform the test or for the lack of a tested reflex; 13 to 18 indicates severe injury, 6 to 12 indicates moderate injury, and 1 to 5 indicates mild injury. Higher scores represent greater injury or severity. On day 1 after MCAO induction, rats that did not show neurological deficits or exhibited mNSS < 6 scores were excluded from the study. The test was performed by an experimenter blinded to the experimental groups, and the average score was recorded.

### Accelerating rotarod performance task

The accelerating rotarod performance task was performed to assess the balance, grip strength, and motor coordination of the MCAO rats. The assessment was conducted on days 1, 4, 7, and 14. Briefly, rats were trained to walk on a rotating rotarod at the speed of 4 to 40 revolutions per minute (rpm), within 300 s, for 3 d (three trials a day with a 15-min inter-trial interval) before ischemic stroke induction. During testing, rats were subjected to the accelerating rotarod while walking using the same parameters employed during training. The test was ended when the rats fell off the rung. The latency to fall from the rotarod apparatus was recorded and the mean value from three trials was determined.

### Quantification of anti-inflammatory and angiogenesis markers

Blood was collected from the retro-orbital plexus under mild anesthesia and subjected to biomarker analysis including transforming growth factor-beta 1 (TGF-β1), interleukin-1 receptor antagonist (IL-1Ra), and vascular endothelial growth factor (VEGF). The analysis conducted was outsourced to Syngene Biology (Bangalore, India) for quantification using an enzyme-linked immunosorbent assay (ELISA) kit. For the assessment of inflammation, TGF-β1 and IL-1Ra were measured at days 4 and 28. TGF-β1 is a multifunctional cytokine that serves as both an immunosuppressive factor and a neuroprotective agent in ischemic stroke (Kinney *et al*. 2018). TGF-β1 is a pleiotropic cytokine that plays a key role in neuroinflammation while IL-1Ra is an endogenous molecule that has been shown to exert potent anti-inflammatory effects in brain injury by inhibiting the pro-inflammatory cytokine, interleukin-1 (IL-1), activity (Glatz *et al*. 2010; Rustenhoven *et al*. 2016). VEGF on the other hand is a potent activator for angiogenesis and neurogenesis (Thau-Zuchman *et al*. 2010). In the present study, the VEGF levels were measured for the assessment of angiogenesis at days 4 and 28.

### Immunohistochemistry assessment of BBB integrity and neurogenesis

We examined the aquaporin-4 (AQP4) expression by IHC analysis, in order to demonstrate the neuroprotective effect of Neuroncell-EX in inhibiting ischemic stroke–induced cerebral edema and BBB breakdown. AQP4 is a transmembrane water channel protein, mainly located at the ischemic peri-focal region and primarily located on the end-feet of astrocytes (Tang *et al*. 2014). The upregulation of AQP4 is closely associated with astrocyte swelling, which subsequently leads to apoptosis of astrocytes and BBB breakdown in the early onset of ischemic stroke (Manley *et al*. 2009), while the neuronal nuclei protein or NeuN is a marker found in the nucleus of mature neurons (Kim *et al*. 2009). Immunohistochemistry (IHC) assessment of AQP4 marker in the brain was evaluated at day 4 while assessment of NeuN was at day 28 after MCAO induction. The earlier timing for AQP4 assessment is to determine whether the hUC-MSCs treatments led to earlier neurological improvement through maintaining BBB integrity (Ramiro *et al*. 2020). On the other hand, NeuN is a marker of neurogenesis or neuron recovery and is not expected to occur in the acute phase but rather in the subacute and chronic phases of stroke. Day 28 assessment is chosen, as by this time, the initial inflammatory responses and cellular changes have stabilized, allowing for a clearer assessment of neuronal integrity and potential recovery.

The animals were euthanized with an overdose of isoflurane at days 4 or 28 for AQP4 and NeuN staining respectively with the required sample size as in Fig. 1*A*. Samples were harvested in 10% neutral buffered formalin for 24 h and paraffin-embedded blocks were made. The tissue block was sectioned at a thickness of 4–5 µm, and the sections were taken onto poly-l-lysine slides. Before proceeding with the staining protocol, the slides were de-paraffinized (using a hot plate and xylene) and rehydrated (in different grades of alcohol). Heat-induced antigen retrieval process was carried out in a two-step process using Tris–EDTA buffer (95 °C for 20 min) and sodium citrate buffer (95 °C for 30 min). Endogenous peroxidase enzyme activity was blocked with a peroxidase-blocking reagent for 3% (10 min). Subsequently, samples were rinsed and washed in phosphate-buffered saline, and incubated with 10% goat serum in 3% bovine serum albumin (BSA) for 60 min in a humidified environment. The tissue sections were incubated with primary antibody at room temperature for 1 h (AQP4—1:500, Proteintech, Rosemont, IL, and NeuN—1:250, Abcam, Cambridge, UK) and at 4 °C overnight. After overnight incubation, the tissue sections were washed and then incubated with a secondary antibody at 1 h at room temperature (1:500 dilutions, goat for AQP4 and NeuN). DAB Chromogen (1 drop of chromogen in 1.5 mL of substrate) for visualizing the intensity of tissue staining. Counterstaining was carried out with Mayer’s hematoxylin (for 1 min) followed by 15 min bluing in running water. All tissue sections were visualized by a light microscope.

### Statistical analysis

All the statistical analyses were performed using GraphPad Prism version 9 for Windows (GraphPad Software, San Diego, CA). The multiple groups comparison was performed using analysis of variance (ANOVA) followed by post hoc Dunnett’s test. When comparing between two groups, we used Student’s *t*-test. Correlation analyses were performed using Pearson’s correlation to determine the relationship between TGF-β1, IL-1Ra, VEGF, AQP4, and NeuN. All data were presented as mean ± standard error of the mean (SEM). The *p*-value of less than 0.05 (*p* < 0.05) was considered statistically significant.

## Results

### Neuroncell-EX is feasible in post-stroke rats

The study design was demonstrated as in Fig. 1*A*. After MCAO/reperfusion surgical procedure, of the 69 rats used, 1 rat was found dead while 68 rats recovered from the surgical procedure within 24 h. Therefore, the cause of death may be due to weakness from the MCAO injury. The dead animal was not replaced. No mortality, adverse event, or tumor formation was observed following IV injection of Neuroncell-EX and throughout the study.

A gradual increase in body weight of all animals was observed (Fig. 1*B*). Initially, a significant lower body weight was observed in stroke control rats from days 1 to 14 post-MCAO, when compared to healthy control rats. Loss of body weight was significantly attenuated in MCAO rats treated with Neuroncell-EX 4 × 10^6^ cells/rat at day 4 compared to stroke control. Of note, the body weight of MCAO rats treated with both doses of Neuroncell-EX (2 and 4 × 10^6^ cells/rat) was higher than stroke control rats throughout day 1 to day 28. Body weight returned to baseline at day 7 post-stroke in all MCAO-treated groups. The total body weight of animals at day 28 was not statistically different between all groups.

### Neuroncell-EX improves functional recovery

**Figure 2.**
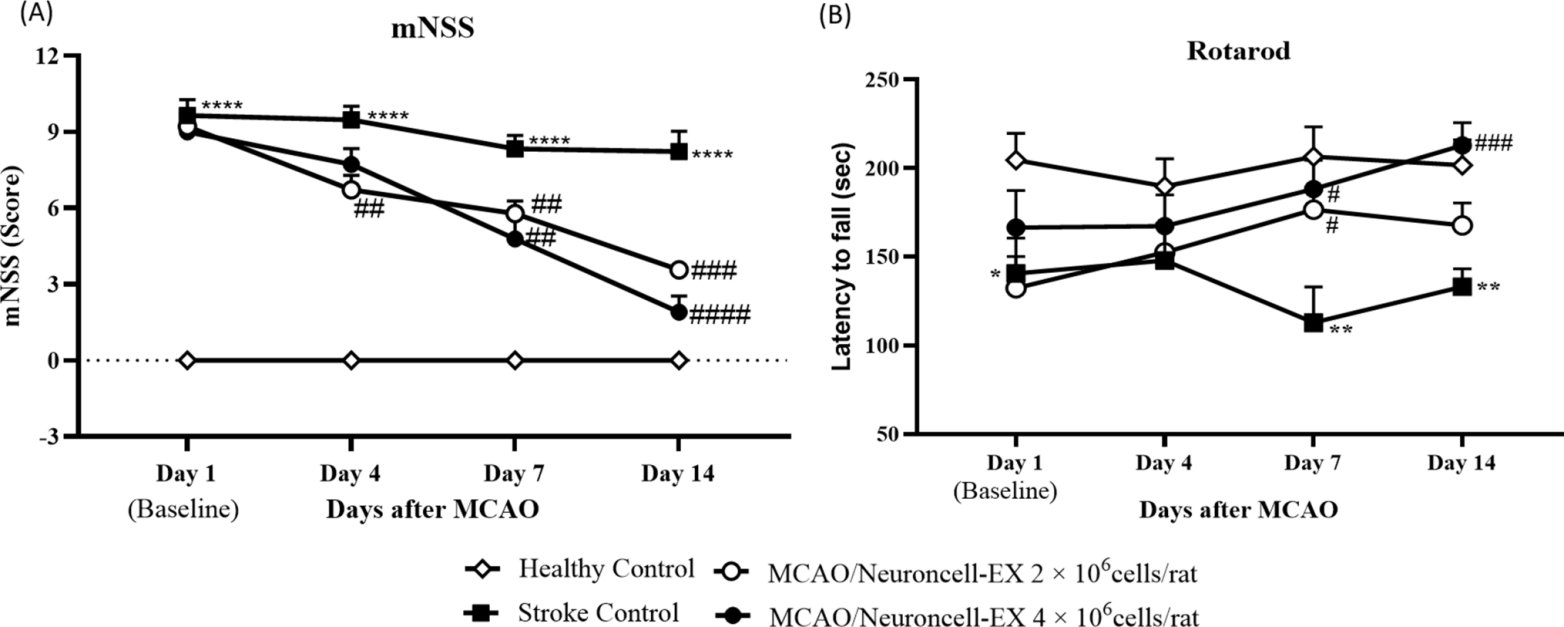
Effects of Neuroncell-EX on the recovery of neurological function in post-stroke rats. Neurological function assessments were performed using (*A*) mNSS and (*B*) rotarod at days 1, 4, 7, and 14 after MCAO induction. Two-way ANOVA followed by post hoc Dunnett’s test was performed to compare multiple groups. Data are presented as mean ± SEM, where * indicates *p* < 0.05, ** indicates *p* < 0.01, and **** indicates *p* < 0.0001 comparing stroke control with healthy control and *#* indicates *p* < 0.05, *##* indicates *p* < 0.01, *###* indicates *p* < 0.001, *####* indicates *p* < 0.0001 comparing Neuroncell-EX-treated groups with stroke control. *Error bars* on each value represent SEM. *MCAO*, middle cerebral artery occlusion; *mNSS*, modified neurological severity score; *SEM*, standard error of the mean.

To evaluate the effects of Neuroncell-EX on functional recovery after stroke in MCAO rats, mNSS and accelerating rotarod performance tasks were conducted on days 1, 4, 7, and 14, which assess neurological functions such as sensory, motor, reflex, and balance, as well as motor function. At day 1 post-stroke (baseline), all MCAO rats exhibited severe neurological deficits prior to initiation of Neuroncell-EX treatment. Based on mNSS test depicted in Fig. 2*A*, the average scores were markedly high in stroke control rats at all time points compared to healthy control (*p* < 0.0001). Lower mNSS represents a better neurological function. A significantly lower mNSS score was observed in MCAO rats treated with Neuroncell-EX 2 × 10^6^ cells/rat as early as day 4 after MCAO compared to stroke control rats (6.7 ± 0.57 versus 9.5 ± 0.51; *p* = 0.0043). The score was also lower for the Neuroncell-EX 4 × 10^6^ cells/rat group but this was not statistically significant. At day 7 after MCAO, mNSS scores were further reduced in MCAO rats treated with Neuroncell-EX 2 × 10^6^ cells/rat and Neuroncell-EX 4 × 10^6^ cells/rat groups compared to stroke control rats (5.8 ± 0.49; *p* = 0.007 and 4.8 ± 0.85; *p* = 0.009 versus 8.3 ± 0.47, respectively). Similarly at day 14, a further neurological improvement was observed in Neuroncell-EX 2 × cells/rat and Neuroncell-EX 4 × 10^6^ cells/rat compared to stroke control rats (3.6 ± 0.29; *p* = 0.0007 and 1.9 ± 0.63; *p* < 0.0001 versus 8.2 ± 0.80, respectively). There is no significant difference between Neuroncell-EX-treated groups at all time points although the mean scores of the Neuroncell-EX 4 × 10^6^ cells/rat group edged closest to healthy control at day 14.

In the accelerating rotarod performance task, the duration time (latency) for rats to fall off the accelerating rod is measured. Longer latency time indicates better motor function. Accelerating rotarod performance task is used to evaluate motor-associated functions such as coordination, balance, and fatigue in rats. As shown in Fig. 2*B*, rotarod performance was poorer in the stroke control rats than in healthy controls as evidenced at days 1 (*p* = 0.04), 7 (*p* = 0.006), and 14 (*p* = 0.004). There was no significant difference in rotarod performance between rats treated with Neuroncell-EX 2 × 10^6^ cells/rat and Neuroncell-EX 4 × 10^6^ cells/rat groups throughout the observation period. At day 7, MCAO rats treated with Neuroncell-EX 2 × 10^6^ cells/rat (176.6 ± 11.02 s; *p* = 0.037) and Neuroncell-EX 4 × 10^6^ cells/rat (188.2 ± 16.34 s; *p* = 0.024) showed significantly higher latency time when compared with stroke control rats (112.8 ± 20.06 s), suggesting a significant improvement in motor function. At 14 days after stroke induction, only MCAO rats treated with Neuroncell-EX 4 × 10^6^ cells/rat (212.9 ± 12.67 s; *p* = 0.0002) displayed better rotarod performance than stroke control rats (133.0 ± 10.06 s).

### Neuroncell-EX attenuates post-stroke inflammation by upregulating the expression of anti-inflammatory cytokines

**Figure 3.**
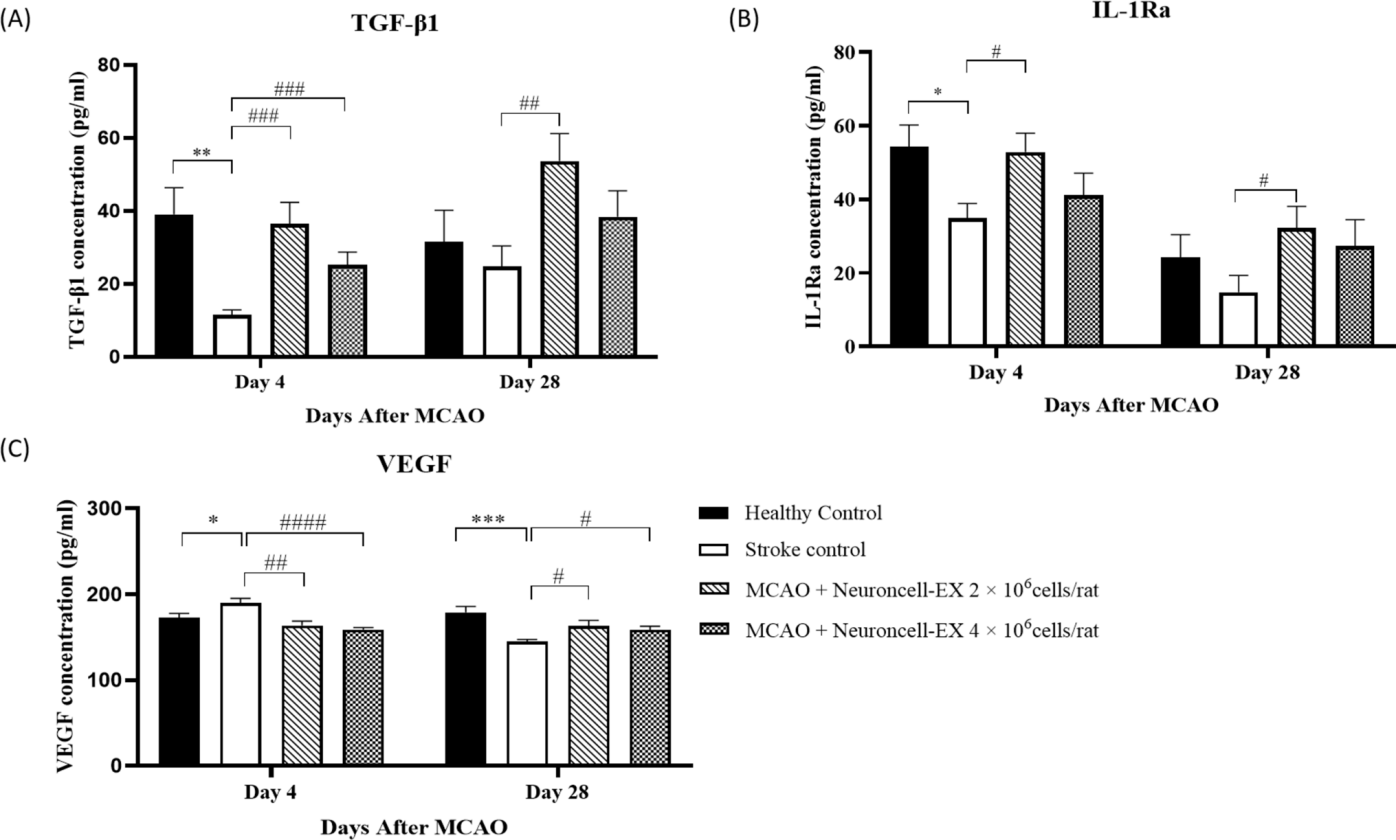
Effect of Neuroncell-EX on the selected markers in serum levels including (*A) *TGF-β1, (*B*) IL-1Ra, and (*C*) VEGF. Data were compared between two groups using Student’s *t*-test. Data are presented as mean ± SEM where * indicates *p* < 0.05, ** indicates *p* < 0.01, and *** indicates *p* < 0.001 comparing stroke control with healthy control and *#* indicates *p* < 0.05, *##* indicates *p* < 0.01, *###* indicates *p* < 0.001, and *####* indicates *p* < 0.0001 comparing Neuroncell-EX-treated groups with stroke control. *IL-1Ra*, interleukin-1 receptor antagonist; *MCAO*, middle cerebral artery occlusion; *SEM*, standard error of the mean; *TGF-β*, transforming growth factor-beta 1; *VEGF*, vascular endothelial growth factor.

In order to determine whether Neuroncell-EX could modulate anti-inflammatory cytokines under ischemic environment, TGF-β1 and IL-1Ra levels were measured in blood serum by ELISA analysis at days 4 and 28 after MCAO induction. TGF-β1 is significantly reduced following MCAO-induced stroke in stroke control compared to healthy control at day 4 (*p* = 0.001) albeit no significant difference observed at day 28. At day 4 after MCAO, serum TGF-β1 levels in MCAO rats treated with Neuroncell-EX 2 × 10^6^ cells/rat (36.4 ± 5.90 ρg/mL; *p* = 0.0004) and Neuroncell-EX 4 × 10^6^ cells/rat (25.3 ± 3.42 ρg/mL; *p* = 0.0007) were significantly higher than stroke control rats (11.4 ± 1.42 ρg/mL) as shown in Fig. 3*A*. However, at day 28, only the Neuroncell-EX 2 × 10^6^ cells/rat group was significantly higher (53.7 ± 7.50 ρg/mL; *p* = 0.006) when compared to stroke control rats (24.8 ± 5.63 ρg/mL).

We also examined the effect of Neuroncell-EX on serum IL-1Ra level in MCAO rats. Significantly lower IL-1Ra was observed in stroke control rats compared to healthy controls (*p* = 0.012) at day 4 but not at day 28. Serum IL-1Ra levels in MCAO rats treated with Neuroncell-EX 2 × 10^6^ cells/rat were significantly higher at days 4 and 28 (52.8 ± 5.15 ρg/mL; *p* = 0.01 and 32.19 ± 5.86 ρg/mL; *p* = 0.03) compared to stroke control rats (34.9 ± 4.00 ρg/mL and 14.7 ± 4.58 ρg/ml) as shown in Fig. 3*B*. There was also an increase in serum IL-1Ra levels in MCAO rats treated with Neuroncell-EX 4 × 10^6^ cells/rat at days 4 and 28 (41.1 ± 6.04 ρg/mL and 27.3 ± 7.22 ρg/mL) compared to stroke control (14.7 ± 4.58 ρg/mL), but these were not statistically significant.

### Effects of Neuroncell-EX on angiogenesis

To elucidate whether Neuroncell-EX exerts any angiogenic effects in the MCAO rat model, serum VEGF levels were investigated at days 4 and 28 after MCAO induction (Fig. 3*C*). As expected, the highest serum VEGF levels were observed in stroke control rats at day 4 (189.6 ± 5.50 ρg/mL; *p* = 0.03) compared to healthy control (172.4 ± 5.35 ρg/mL) indicating acute and severe ischemic injury. Conversely, the VEGF levels of stroke control were significantly lower at day 28 (144.8 ± 2.63 ρg/mL; *p* = 0.0002) when compared to the healthy control rats (178.9 ± 6.70 ρg/mL), indicating the lack of longer-term angiogenic stimulation. Similarly, serum VEGF levels in MCAO rats treated with Neuroncell-EX 2 × 10^6^ cells/rat and Neuroncell-EX 4 × 10^6^ cells/rat were significantly lower at day 4 compared to stroke control (163.48 ± 5.35 ρg/mL; *p* = 0.002 and 158.85 ± 2.30 ρg/mL; *p* < 0.0001 versus 189.6 ± 5.50 ρg/mL, respectively). The levels were significantly higher than stroke control at day 28 when treated with Neuroncell-EX 2 × 10^6^ cells/rat and Neuroncell-EX 4 × 10^6^ cells/rat (163.20 ± 6.52 ρg/mL; *p* = 0.017 and 158.42 ± 4.39 ρg/mL; *p* = 0.016 versus 144.8 ± 2.63 ρg/mL, respectively).

### Effects of Neuroncell-EX on blood–brain barrier (BBB) integrity

**Figure 4.**
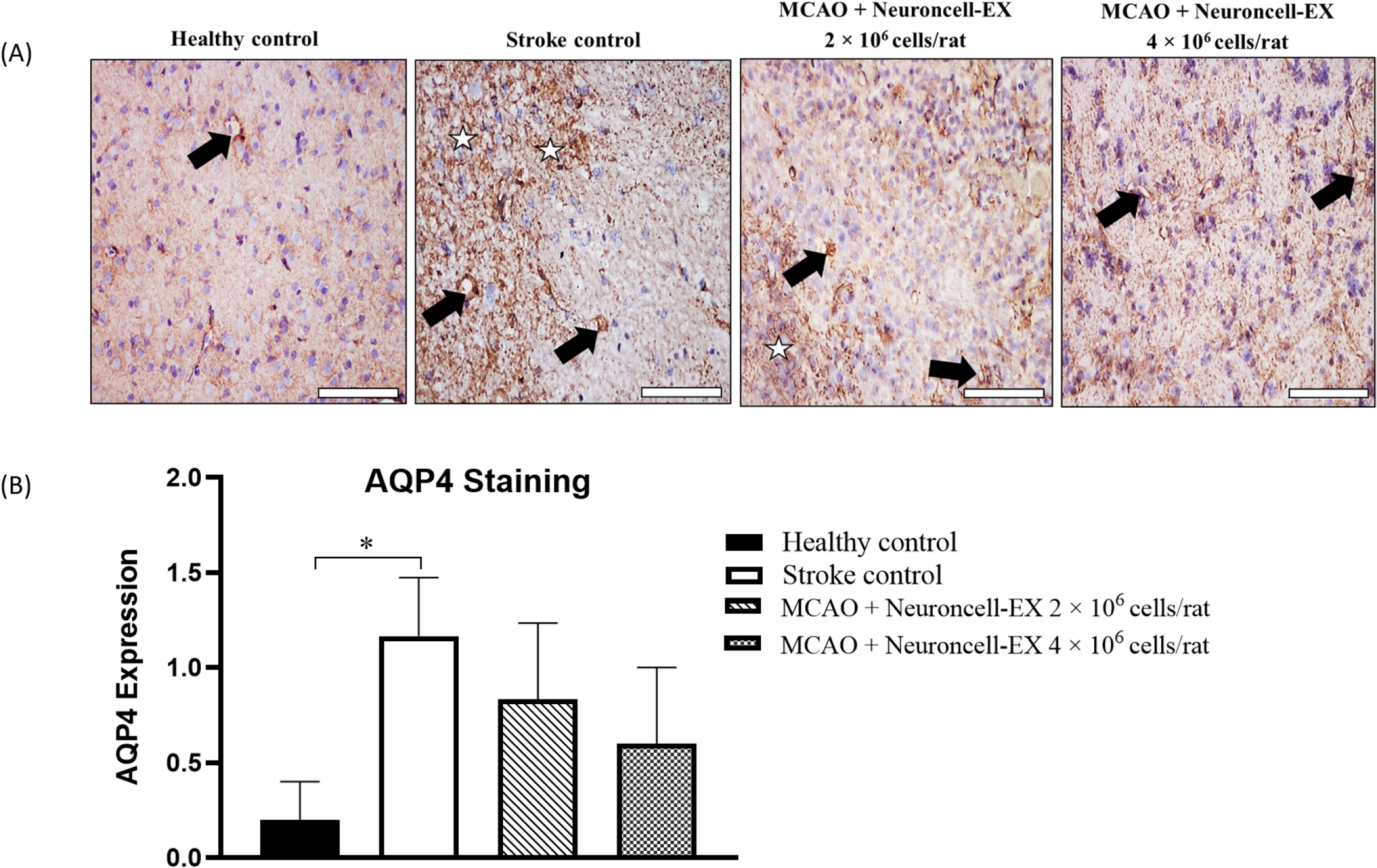
Effect of Neuroncell-EX on the expression of AQP4 in rats across all four groups. (*A*) Representative images of AQP4 immunostaining analysis in the rat brain sections at day 4 following MCAO. AQP4-positive expression is stained with *brown* in the neuropil (marked by a *white star*) and perivascular areas (marked by a *black arrow*). The *scale bars* represent 200 μm. (*B*) The scores of AQP4-positive expression were compared between two groups using Student’s *t*-test. Data are presented as mean ± SEM where * indicates *p* < 0.05 comparing stroke control and healthy control groups. *AQP4*, aquaporin-4; *MCAO*, middle cerebral artery occlusion.

AQP4 expression was investigated by IHC staining at day 4 post-MCAO and revealed to be upregulated in the stroke control rats, as evidenced by intense expression in the neuropil and perivascular areas of the cortex and striatum regions (Fig. 4*A*), and this expression in stroke control was significantly higher (1.17 ± 0.31; *p* = 0.033) as compared to that in healthy control rats (0.20 ± 0.20) (Fig. 4*B*). Treatment with both doses of Neuroncell-EX showed lower expression of AQP4 in the cortex and striatum compared to stroke control albeit not statistically significant.

### Effects of Neuroncell-EX on neurogenesis

**Figure 5.**
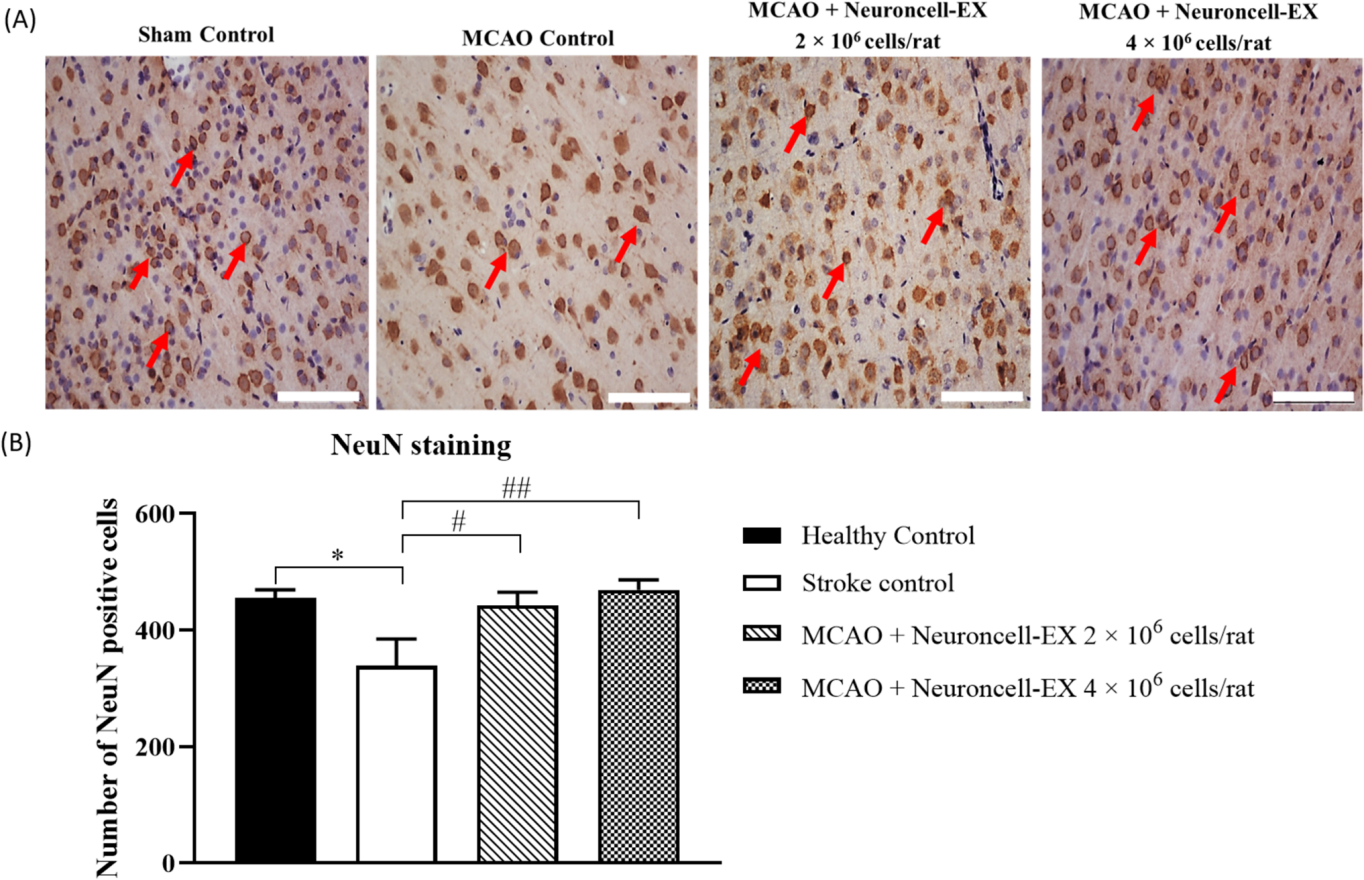
Effect of Neuroncell-EX on the expression of neuronal protein marker NeuN in the MCAO rat model. (*A*) Representative images of NeuN immunostaining analysis in the rat brain sections at day 28 following MCAO. NeuN-positive expression were stained with *brown*, marked by *red arrow*. The *scale bars* represent 200 μm for micrographs. (*B*) Comparison of the mean number of NeuN-positive cells in high power field (magnification 200 ×) from 4 random fields across groups. One-way ANOVA followed by post hoc Dunnett’s test was performed for comparison of multiple groups. Data are presented as mean ± SEM, where * indicates *p* < 0.05 comparing stroke control to healthy group and *#* indicates *p* < 0.05, *##* indicates *p* < 0.01 comparing Neuroncell-EX-treated groups to stroke control. *SEM*, standard error of the mean; *MCAO*, middle cerebral artery occlusion.

NeuN was investigated by IHC staining at day 28, and the number of NeuN-positive cells was quantified in four different fields. As shown in Fig. 5*A*, the dynamic changes in the number of NeuN-positive cells were observed in rats, following MCAO induction. The histological image and the graph showed that the number of NeuN-positive cells was significantly decreased in the cortex of stroke control rats when compared to healthy control (338.80 ± 45.90 versus 455.40 ± 13.74; *p* = 0.022). While both treatments with 2 × 10^6^ cells/rat and 4 × 10^6^ cells/rat of Neuroncell-EX have significantly higher expression of NeuN compared to stroke control at day 28 (442.50 ± 22.65; *p* = 0.043 and 469.30 ± 16.99; *p* = 0.008 versus 338.80 ± 45.90, respectively) as shown in Fig. 5*B*.

### Correlation between TGF-β1, IL-1Ra, VEGF, AQP4, and NeuN

Pearson’s correlation analyses were performed between values for different markers at day 4 by pooling together data from all four groups to determine their association. A positive but weak correlation was observed between TGF-β1 and VEGF (*r* = 0.324, *p* = 0.030). Conversely, an inverse correlation was observed between TGF-β1 and AQP4 (*r* = − 0.412, *p* = 0.024). However, there were no significant associations between any other markers.

## Discussion

In the present work, we have demonstrated the feasibility and therapeutic efficacy of a single IV administration of human umbilical cord–derived mesenchymal stem cell (Neuroncell-EX) in a rat model of acute ischemic stroke. We had chosen the intravenous approach as it would be less invasive. The MCAO model with intraluminal suture is a “gold standard” and clinically relevant to validate focal cerebral ischemia in humans. In this technique, the blood supply to the MCA is interrupted by inserting a suture from the external carotid artery into the internal carotid artery until the tips occlude the origin of the middle cerebral artery (Fluri *et al*. 2015). This model is characterized by extremely large infarct size, cerebral edema, impaired recovery processes, and motor dysfunction, which is similar to the phenomenon following large territorial infarct in humans (Cai *et al*. 2016; Sicard & Fisher 2009). In addition, most of the previous studies applied this model as it is more clinically relevant compared to other models. This model would be appropriate in demonstrating the effect of our hUC-MSCs as it is less invasive, requires no craniectomy procedure, and particularly, mimics the ischemic stroke in humans (Fluri *et al*. 2015). Only 1 rat died following MCAO induction. No mortality, adverse events, or tumor formation was observed in the MCAO rats following IV injection of Neuroncell-EX at either dosage throughout the study which aligned with our previous safety dose range finding study (Chin *et al*. 2024).

Several rat animal model studies have suggested that early administration of MSCs in the acute phase between 3 h and 3 d after stroke may help improve neurological function (Wang *et al*. 2014; Tobin *et al*. 2020). In this study, Neuroncell-EX was administered 24 h after MCAO induction which is equivalent to 30 d in humans (Sengupta 2013). Functional recovery is often a key measure of stroke improvement (Schaar *et al*. 2010), which we were able to assess through functional and behavioral tests in the rat. Neuroncell-EX at both doses led to significant functional recovery and improvement from day 7 to day 14 post-stroke approaching the mNSS scores of healthy controls, while the stroke control group did not demonstrate any improvements. The Neuroncell-EX 2 × 10^6^ cells/rat group showed earlier improvement in mNSS score at day 4 but was overtaken by the Neuroncell-EX 4 × 10^6^ cells/rat group by days 7 and 14. The scores between both treatment groups were not statistically different at all time points. In the rotarod test, both treatment groups showed significant motor function improvement compared to stroke control at day 7 but only the 4 × 10^6^ cells/rat group remained statistically different by day 14. These findings suggest that while treatment with a dose of 2 × 10^6^ cells/rat may lead to earlier functional improvement, it is treatment with the higher dose of 4 × 10^6^ cells/rat that leads to more sustained improvements in the longer follow-up. The initial advantage seen in the Neuroncell-EX 2 × 10^6^ cells/rat group maybe due to its anti-inflammatory effects. Neuroncell-EX dose at 2 × 10^6^ cells/rat showed significant elevation of TGF-β1 and IL-1Ra levels at days 4 and 28, following MCAO induction, with levels approaching healthy controls. Neuroncell-EX dose at 4 × 10^6^ cells/rat showed significant elevation of TGF-β1 level at day 4 only.

In the past few years, several preclinical and clinical studies have proved the application of MSCs in promoting functional recovery through immunomodulatory and anti-inflammatory actions (Marei *et al*. 2018; Li *et al*. 2021a, b). Neuroinflammation plays a crucial role in the pathogenesis of ischemic stroke (Jin *et al*. 2010). Following ischemic stroke, reactive oxygen species (ROS) and pro-inflammatory mediators, such as cytokines and chemokines, are secreted from damaged brain tissue, leading to the activation of resident inflammatory cells such as astrocytes and microglia. Upon activation, these resident inflammatory cells secrete pro-inflammatory cytokines, chemokines, and matrix metalloproteinases (MMPs) which further contribute to the disruption of the BBB and infiltration of leukocytes from the blood into the brain tissue, ultimately exacerbating the penumbra and surrounding areas (Jin *et al. *2010; Dabrowska *et al*. 2019; Guo *et al*. 2021). In the present study, cytokine analysis was performed on days 4 and 28, which are in the transition from the acute to the sub-acute phase of stroke. As reported in previous findings, the immune response shifts from pro-inflammatory to anti-inflammatory states in the subacute phase of stroke (Noh *et al*. 2020). Therefore, we determined the immunomodulatory and anti-inflammatory actions of Neuroncell-EX through the regulation of TGF-β1 and IL-1Ra. TGF-β1 plays an important role in angiogenesis, inflammatory reactions, neuronal survival, neurogenesis, and preventing BBB disruption (Ma *et al*. 2008; Zhu *et al*. 2022). TGF-β1 also regulates the synthesis of angiogenic VEGF-A (Nakagawa *et al*. 2004) and expresses VEGF under hypoxic conditions (Nam *et al*. 2010). IL-1Ra is also crucial in the regulation of post-ischemic inflammation, and its expression is rapidly upregulated in response to inflammation and hypoxia (Oh *et al*. 2018). IL-1Ra could also act as a mediator and blocks the action of IL-1 and inhibits AQP4 expression in the ischemic brain (Oh *et al*. 2018). Here, our findings were in agreement with previous studies that found upregulation of IL-1Ra (Clausen *et al*. 2016; Oh *et al*. 2018) and TGF-β1 (Cheng *et al*. 2015; Moisan *et al*. 2016).

Our study also explored the other possible neuroprotective mechanisms of Neuroncell-EX including angiogenesis (VEGF), maintenance of BBB integrity (AQP4 expression), and neurogenesis (NeuN expression) in the rat model of ischemic stroke. VEGF enhances angiogenesis in the ischemic brain which in turn reduces neurological deficits during stroke recovery (Zhang *et al.* 2000; Matsuo *et al*. 2013). Angiogenesis is one of the important processes that may contribute to repair brain ischemic injury via the formation of new microvessels in the brain after ischemic stroke (Merino-González *et al*. 2016). In several studies, MSCs were able to promote vascularization, stimulate angiogenesis, and secrete various molecules including VEGF and other growth factors (Merino-González *et al*. 2016). Several studies also reported that hUC-MSCs are able to promote angiogenesis in stroke animal models evidenced by the regeneration of vascular endothelial cells, increase in newly formed blood vessels, improved vascular density and perimeter, and enhanced cerebral microvascular perfusion (Lin *et al*. 2011; Zhao *et al*. 2015). In the present study, both doses of Neuroncell-EX significantly increased VEGF levels at day 28 in MCAO-treated rats compared to stroke control. On the other hand, at day 4, the VEGF expression in the stroke control group is significantly higher than healthy control and both treatment groups. The same phenomenon was observed in previous clinical studies that showed VEGF levels in stroke patients increased immediately after stroke onset and persisted for at least 3 mo (Matsuo *et al*. 2013). This is likely a compensatory mechanism against ischemic injury in the acute stages. However, VEGF expression is also a pathogenic factor in stroke, particularly in vascular permeability. Overexpression of VEGF following ischemic injury induced BBB breakdown and vascular leakage in the acute ischemic brain, leading to cerebral edema that can exacerbate the ischemic brain injury (Chen *et al*. 2021; Sun *et al*. 2022). Zhang *et al*. demonstrated that early administration of VEGF 1 h after stroke exacerbated BBB leakage, increased hemorrhagic transformation, and induced ischemic cell injury, whereas administration of VEGF at 48 h after stroke promoted angiogenesis and functional recovery. Their findings illustrated how VEGF might cause BBB leakage in the early onset stroke but enhanced angiogenesis in the late phase (Chen *et al*. 2021). Thus, it is desirable to have lower VEGF expression at day 4. By day 28, Neuroncell-EX may exert its angiogenic effect by upregulating serum VEGF levels higher than stroke control, and eventually lead to neurogenesis and functional recovery. Taken together, these findings suggest that Neuroncell-EX treatment reduces the initial brain injury and stimulates subsequent angiogenesis as demonstrated by the initial decrease and subsequent increase in serum VEGF levels after stroke.

Next, we determined the protective effect of Neuroncell-EX towards BBB permeability and cerebral edema injury. There is growing evidences that inflammation plays an important role in blood–brain barrier (BBB) breakdown and cerebral edema (Jin *et al.* 2010; Dabrowska *et al*. 2019). AQP4 is a transmembrane water channel protein, located mainly at the end-feet of astrocytes (Thi *et al*. 2008). The upregulation of AQP4 is closely related to astrocyte swelling, which subsequently leads to apoptosis of astrocytes and BBB collapse in the early onset of ischemic stroke (Manley *et al*. 2009). Inhibition of AQP4 has been found recently as therapy targeting central nervous system edema post-injury, including attenuating cerebral edema associated with ischemic stroke and eventually promoting neurological recovery (Sun *et al*. 2022). In the present study, we found significant upregulation of AQP4 expression, mainly located in the striatum and cortex of stroke control rats compared to the healthy control. However, our results showed a lower expression of AQP4 on day 4 in rats treated with both doses of Neuroncell-EX compared to the stroke control, but the difference was not statistically significant.

Finally, we postulated the effect of Neuroncell-EX in promoting neurogenesis and preventing neuron apoptosis via upregulation of NeuN expression in an ischemic stroke model. NeuN is a useful protein marker that is localized in nuclei and perinuclear cytoplasm of most neurons that are widely used in neuropathological studies to differentiate neurons from glial cells and commonly expressed in the mature postmitotic neurons (Ünal-Çevik *et al*. 2004; Lavezzi *et al*. 2013; Gusel’nikova & Korzhevskiy 2015). NeuN staining was performed on day 28 after hUC-MSCs administration as any immature neurons would have already reached their maturation phase within 2–4 wk, as reported in previous studies (Radic *et al*. 2017). A significantly lower NeuN expression was observed in stroke control compared to healthy control. A lower expression of NeuN may indicate neuronal degradation, loss of cellular morphology without neuronal death (Ünal-Çevik *et al*. 2004; Lancaster & Dalmau 2012), or with neuronal apoptosis (Davoli *et al*. 2002). In the present study, the treatment with Neuroncell-EX at both doses significantly increased NeuN expression at day 28. Our results were consistent with previous findings that showed a higher number of NeuN cells in hUC-MSCs-treated groups (Li *et al*. 2023a, b; Shen *et al*. 2023).

hUC-MSCs therefore upregulate TGF-β1 and IL-1Ra, and these anti-inflammatory factors may interact indirectly with the endogenous system, increase the efficiency of neurotrophic factors, and modulate their action, subsequently enhancing the neurogenesis in the stroke brain. Various neural progenitors have demonstrated significant involvement in region-specific angiogenesis within the brain through the signaling pathway of TGF-β1 (Zhang & Yang 2020). In addition to its anti-inflammatory activity, TGF-β1 controls cell growth, proliferation, and apoptosis (Battista *et al*. 2006) and induces VEGF secretion, a key mechanism in neurogenesis (Nakagawa *et al*. 2004; Nam *et al*. 2010). A study by Ma and co-workers also found increased numbers of NeuN-positive cells in the subventricular zone and striatum region following TGF-β1 treatment in a stroke animal model (Ma *et al*. 2008). We have demonstrated a modest correlation of the markers indicating the association between anti-inflammation with angiogenesis and maintaining BBB integrity. Of course, additional in vitro and in vivo studies are needed to further validate these associations.

One of the limitations of this study was the age disparity between animal models and humans, which may affect the translation of some results to the clinical setting. In addition, as our current analysis focused on functional improvement, the measurement of infarct size was not evaluated. Due to insufficient sample size, we also could not assess pro-inflammatory cytokines, other cognitive assessments, and additional staining analyses to support the effect of Neuroncell-EX in promoting angiogenesis and neurogenesis.

## Conclusion

Our findings demonstrate that treatment with a single IV administration of Neuroncell-EX at 24 h following ischemic stroke is feasible, demonstrated significant functional improvements, and restored the number of neurons to near-normal levels (neurogenesis) in the MCAO rats. In addition, Neuroncell-EX also reduced cerebral and systemic inflammation by regulating TGF-β1 and IL-1Ra levels following ischemic stroke. Neuroncell-EX treatment is associated with immunomodulation, angiogenesis, and neurogenesis by regulating TGF-β1, IL-1Ra, VEGF, and NeuN. Overall, Neuroncell-EX is feasible and effective at both doses. While the dose of 2 × 10^6^ cells/rat provided earlier functional recovery and sustained anti-inflammation, the higher dose of 4 × 10^6^ cells/rat resulted in eventual better functional recovery based on mNSS and rotarod tests. The equivalent dosage and time interval in humans can now be applied when designing clinical trials.

## Data Availability

The data analyzed to support the findings of this study are available from the corresponding author upon request.
